# *PTPN22* 1858C>T Polymorphism Distribution in Europe and Association with Rheumatoid Arthritis: Case-Control Study and Meta-Analysis

**DOI:** 10.1371/journal.pone.0024292

**Published:** 2011-09-16

**Authors:** Michele Ciro Totaro, Barbara Tolusso, Valerio Napolioni, Francesca Faustini, Silvia Canestri, Alice Mannocci, Elisa Gremese, Silvia Laura Bosello, Stefano Alivernini, Gianfranco Ferraccioli

**Affiliations:** 1 Division of Rheumatology, Catholic University of the Sacred Heart, Rome, Italy; 2 Laboratory of Human Genetics, School of Biosciences and Biotechnologies, University of Camerino, Camerino, Italy; 3 Unit of Hygiene, Department of Public Health and Infectious Diseases, Sapienza University of Rome, Rome, Italy; South Texas Veterans Health Care System, United States of America

## Abstract

**Objective:**

The *PTPN22 rs2476601* polymorphism is associated with rheumatoid arthritis (RA); nonetheless, the association is weaker or absent in some southern European populations. The aim of the study was to evaluate the association between the *PTPN22 rs2476601* polymorphism and RA in Italian subjects and to compare our results with those of other European countries, carrying out a meta-analysis of European data.

**Methods:**

A total of 396 RA cases and 477 controls, all of Italic ancestry, were genotyped for *PTPN22 rs2476601* polymorphism. Patients were tested for autoantibodies positivity. The meta-analysis was performed on 23 selected studies.

**Results:**

The *PTPN22* T1858 allele was significantly more frequent in RA patients compared to controls (5.7% *vs.* 3.7%, *p* = 0.045). No clear relationship arose with the autoantibodies tested. The 1858T allele frequency in Italian RA patients was lower than the one described in northern European populations and similar to the frequency found in Spain, Turkey, Greece, Tunisia. A clear-cut North-South gradient arose from the analysis.

**Conclusions:**

The *PTPN22* T1858 allele is associated with RA in the Italian population. A North-South gradient of the allele frequency seems to exist in Europe, with a lower prevalence of the mutation in the Mediterranean area.

## Introduction

Genetic factors are thought to be responsible for up to 50–60% of the rheumatoid arthritis (RA) liability [1]. The minor allele (T) at 1858C>T (*rs2476601*) single-nucleotide polymorphism (SNP) in the protein tyrosine phosphatase non-receptor type 22 (*PTPN22*, gene map locus 1p13) gene has been extensively associated with susceptibility to various autoimmune diseases [2]. The *rs2476601* determines a R620W substitution resulting in a gain-of-function form of the enzyme Lyp (encoded by the *PTPN22* gene), thus leading to a stronger suppression of early T cell activation process [3]. The B cell compartment seems altered by this SNP as well [4]. The association of the *PTPN22* 1858C>T SNP with RA is well established among many populations all over the world, especially in anti-CCP (cyclic citrullinated peptides) antibodies positive RA patients [5]. However, a weaker or a complete lack of association has been reported in some southern European populations [6]–[9]. A review of the literature suggests a lower frequency of the T1858 allele in RA patients of the Mediterranean area. According to Orozco *et al.*, there was no association of *PTPN22* 1858C>T SNP with early RA in Spain [6]. In Turkey, Sahin *et al.* found no association of the 1858C>T SNP with RA [7]. Moreover, a lack of association has also been reported in a Tunisian and in a Greek population [8], [9].

Although the role of *PTPN22 rs2476601* SNP in autoimmunity and its association with RA are undoubted, as recently confirmed by genome-wide association analyses [10], it is important to take into account geographical and anthropological differences when performing genetic epidemiology studies.

To date, no data are available regarding the possible association between *PTPN22* 1858C>T polymorphism and rheumatoid arthritis in patients of Italic ancestry. The aims of the study were: 1. To evaluate the *PTPN22 rs2476601* SNP distribution in an Italian cohort; 2. To define, by means of a systematic review and meta-analysis, the association between the *PTPN22* 1858C>T polymorphism distribution in Europe and rheumatoid arthritis.

## Methods

### Ethical statement

The ethical approval for the study was obtained from the Catholic University of the Sacred Heart Ethical Committee. All subjects gave their written informed consent on the analysis of the *PTPN22* gene polymorphism and autoantibodies testing.

### Case-control study

#### Population and setting

Cases were recruited from the Division of Rheumatology of the Catholic University of the Sacred Heart of Rome. Patients fulfilled at least four of the American College of Rheumatology criteria for RA [11]. When looking back at the database with characteristics of each patient, all satisfied the 2010 ACR criteria as well [12]. The controls sample includes healthy subjects matched for age, sex and geographical origin with case subjects.

In order to calculate the sample size, the following parameters were used: power 80%, level of confidence 95%, estimated frequency of *PTPN22* T1858 allele in controls of 8.9% and of 15.1% in RA patients (based on the mean value of the European studies). Sample size was estimated to be: 462 cases and 462 controls. Patients were recruited consecutively between January 2008 and December 2010 from the Division of Rheumatology of the Catholic University of the Sacred Heart of Rome.

All patients' sera were tested for the presence of anti-CCP, IgM RF (rheumatoid factor) and IgA RF autoantibodies (ELISA method, Axis-Shield Diagnostics, Dundee, UK for anti-CCP and Orgentec diagnostika, Mainz, Germany for IgM and IgA RFs).

#### Genotyping

Genomic DNA was isolated from whole blood through FlexiGene DNA kit (Qiagen, Valencia, CA) according to the manufacturer instructions.

The *PTPN22* 1858C>T SNP was determined by the restriction fragment length polymorphism-polymerase chain reaction (PCR) based method as previously described [13], in all the patients and controls. Briefly, oligonucleotides 5′-TCACCAGCTTCCTCAACCACA-3′ and 5′-GATAATGTTGCTTCAACGGAATTT-3′ were used as primers for *PTPN22* 1858C>T SNP. The C→T transition at codon 620 creates in the T1858 allele a restriction site for XcmI (New England Biolabs, Beverly, MA. USA). The product of PCR was digested with XcmI at 37°C for 3 hours and each digestion was resolved on 3% agarose gel (Figure S1). Repeated typing was performed in 10% of patient samples, with identical results in all cases.

### Meta-analysis

#### Identification of eligible studies and data extraction

The electronic medical databases used for the search were Pubmed, Embase and the Cochrane Library. In the research, we used the keywords: “arthritis”, “rheumatoid”, “*PTPN22*”, “polymorphism” applying the following algorithm: (*PTPN22* OR “protein tyrosine phosphatase non-receptor type 22” OR Lyp) AND (rheumatoid AND/OR arthritis) AND (polymorphism OR SNP). The identification of eligible studies was carried out from 2000 until December 2010 and it was not restricted to English language. Studies references were also analyzed to find any study not available from the electronic databases. A study was included in the systematic extraction of the data if: 1. it was published before 2011; 2. it was about European patients with rheumatoid arthritis; 3. *PTPN22* 1858C>T SNP was evaluated and genotypes data were clearly expressed; 4 it was a case-control study; 5. it was not a transmission disequilibrium test in which family members were studied. Data related to the *PTPN22* 1858C>T SNP in RA patients and controls groups were extracted to perform the meta-analysis. Data extraction and quality assessment, according to a score sheet available for observational studies [14], were performed independently by two different investigators.

### Statistical analysis

Data regarding the *PTPN22* 1858C>T SNP in our RA patients and controls groups were checked for deviation from Hardy-Weinberg equilibrium (χ^2^ test). Descriptive statistics was performed using frequencies and percentages. The association between alleles and genotypes with RA was investigated applying Exact Fisher's Test, and calculating Odds Ratio (OR) with 95% Confidence Interval (95%CI). Statistical analysis was performed with SPSS 19.0 software for Windows.

Three different meta-analyses were carried out using StatDirect statistical software Version2.7.8. The first one evaluated the association of *PTPN22* 1858C>T SNP with RA considering all published studies, present study included; the second one presented data excluding the Italian population; the third one considered the studies with a quality score ≥11, that corresponds to the median quality score.

Forest-plots graphs were produced in order to estimate the pooled association between the *PTPN22* 1858C>T SNP and RA. The Cochran's Q test was performed to evaluate studies heterogeneity, thus using the random effect model when the test highlighted differences between studies and the fixed effect model when no significant differences were shown. Publication bias was quantified by inspection of funnel plot and computation of Egger and Begg test probability values [15]. Significance threshold was set at *p*<0.05 (2-tailed) for all analyses.

## Results

### *PTPN22* 1858C>T SNP in Italy

The studied sample was composed of 396 RA patients and 477 controls (the power of the study was 75% for the cases). The genotype distribution of the *PTPN22* 1858C>T SNP was in Hardy-Weinberg equilibrium in both groups. Seventy-nine percent of the RA patients were female, 67% were positive for anti-CCP antibodies, 50% were positive for IgM RF (rheumatoid factor), and 34% were positive for IgA RF. Moreover, 74% were positive for at least one of the autoantibodies tested.

In our center, the analysis of RA patients and controls, all of Italic ancestry, showed a *PTPN22* T1858 allele frequency of 5.7% in patients compared to 3.7% in controls (OR = 1.58; 95%CI = (1.01–2.49); *p* = 0.045) (Table 1). The frequency of positivity for anti-CCP antibodies tended to be higher in RA patients carrying the T allele (79.5%) compared to subjects with C/C genotype (65.6%; *p* = 0.09). No difference was detected in the percentage of IgA and IgM RFs positivity between RA patients carrying the T allele and patients with C/C genotype (data not shown).

**Table 1:** Analysis of the association of *PTPN22* 1858C>T SNP with RA patients and healthy subjects.

		Genotype						
	Group	C/C	C/T	T/T	T Allele	HWE (*p*)^1^	OR (95%CIs)^2^	Allelic *p* 3
**Females**	RA patients (n = 313) (mean age: 55.7±13.7)	276 (91.6%)	36 (11.5%)	1 (0.3%)	38 (6.1%)	*1.00*	**1.56 (0.95–2.55)**	*0.074*
	Healthy controls (n = 377) (mean age: 55.5±14.1)	348 (92.3%)	28 (7.4%)	1 (0.3%)	30 (4.0%)	*0.45*		
**Males**	RA patients (n = 83) (mean age: 59.7±12.2)	76 (91.6%)	7 (8.4%)	0 (0.0%)	7 (4.2%)	*1.00*	**1.72 (0.54–5.52)**	*0.359*
	Healthy controls (n = 100) (mean age: 59.4±12.0)	95 (95.0%)	5 (5.0%)	0 (0.0%)	5 (2.5%)	*1.00*		
**All**	RA patients (n = 396) (mean age: 56.6±13.4)	352 (88.9%)	43 (10.9%)	1 (0.2%)	45 (5.7%)	*1.00*	**1.58 (1.01–2.49)**	***0.045***
	Healthy controls (n = 477) (mean age: 56.3±14.0)	443 (92.9%)	33 (6.9%)	1 (0.2%)	35 (3.7%)	*0.48*		

1 HWE: Hardy-Weinberg equilibrium.

2 Odds ratios expressed as carriers of T1858 allele *vs.* non-carriers considering healthy controls as control group.

3 Fisher's exact test of odds ratios.

### Meta-analysis of the reported studies regarding the *PTPN22* 1858C>T SNP in Europe

The association of the *PTPN22* 1858C>T SNP with RA in Europe was found in 23 studies [5], [7], [8], [16]–[35] (see PRISMA Checklist S1 and Figure S2). The characteristics of each study are shown in Table 2.

**Table 2:** Characteristics of the selected studies (n = 24) concerning the association between *PTPN22* 1858C>T SNP and RA in Europe.

Study	Ref.	Year	Country	Quality	RA C/T-T/T	Controls C/T-T/T	RA total	Controls total
Seldin *et al.*	16	2005	Finland	10	372	406	1030	1400
Plenge *et al.*	17	2005	Sweden	11.5	432	203	1513	874
Hinks *et al.*	18	2005	UK	11	289	114	886	595
Steer *et al.*	19	2005	UK	10	84	62	302	374
Wesoly *et al.*	20	2005	Netherlands	10	93	155	416	891
Zhernakova *et al.*	21	2005	Netherlands	10	42	88	151	528
Orozco *et al.*	22	2005	Spain	11.5	163	146	826	1036
Johansson *et al.*	5	2006	Sweden	12	35	71	89	360
Harrison *et al.*	23	2006	UK	10	179	109	686	566
Pierer *et al.*	24	2006	Germany	11.5	148	67	390	349
Viken *et al.*	25	2007	Norway	10.5	264	119	861	557
Lie *et al.*	26	2007	Norway	12.5	75	119	221	555
Kokkonen *et al.*	27	2007	Sweden	12	166	209	504	970
Majorczyk *et al.*	28	2007	Poland	11	61	118	173	543
Wesoly *et al.*	29	2007	Netherlands	10.5	183	55	661	284
Eike *et al.*	30	2008	Norway	11	213	191	686	952
Farago *et al.*	31	2008	Hungary	11	158	24	399	107
Starck *et al.*	32	2009	Slovakia	12.5	158	63	514	302
Sahin *et al.*	7	2009	Turkey	10.5	11	9	167	177
Chabchoub *et al.*	8	2009	Tunisia	11	7	12	150	236
Sfar *et al.*	33	2009	Tunisia	11	33	2	133	100
Morgan *et al.*	34	2009	UK	10	1324	705	4789	3630
Majorczyk *et al.*	35	2010	Poland	11	259	118	371	543
Present study	-	2011	Italy	-	44	34	396	477

**Figure 1:**
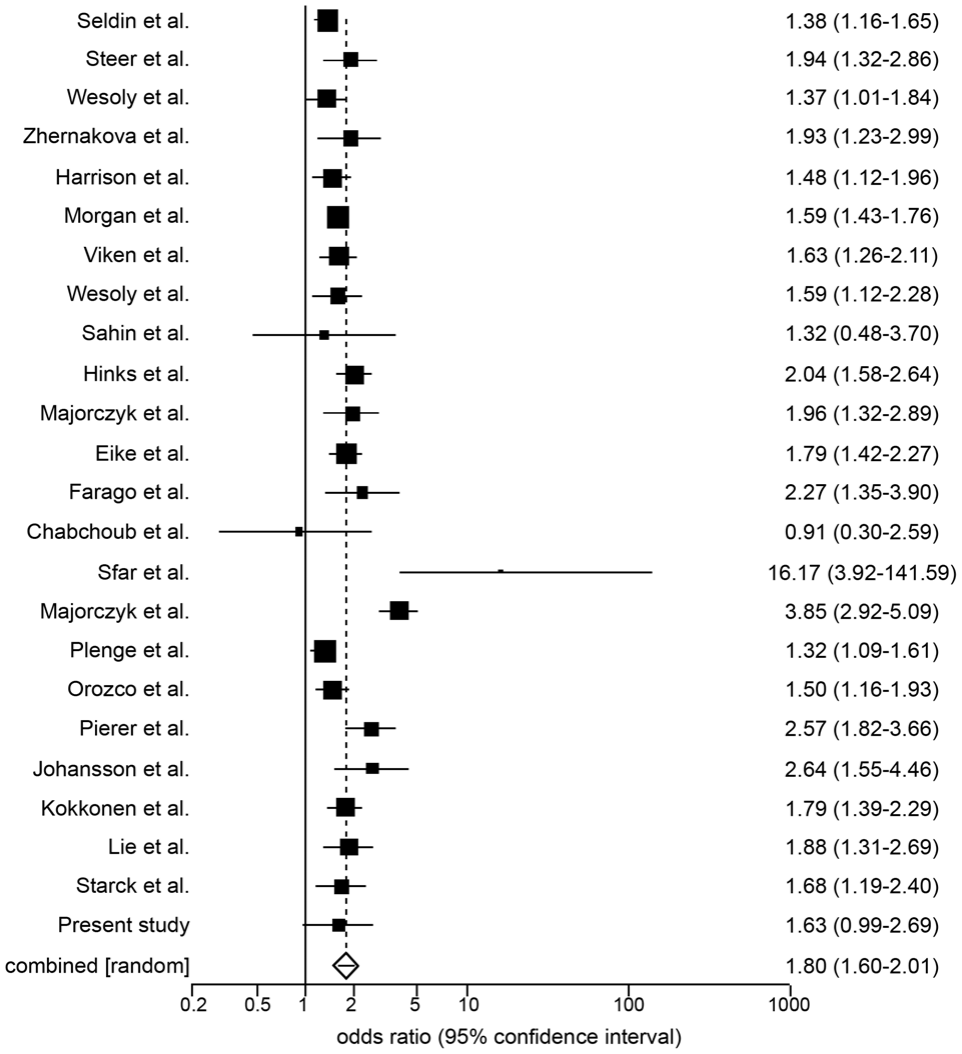
Forest plot of the first meta-analysis. Forest plot of published studies in relation to the first meta-analysis (24 studies). The association of the *PTPN22* 1858C>T SNP with RA was evaluated through the Odds ratios measures. The random effect model was used.

In the first meta-analysis a significant and positive association between the *PTPN22* 1858C>T SNP and RA was found: pooled OR = 1.79 with 95%CI = (1.60–2.01) (Figure 1). The Cochran's Q test established the presence of heterogeneity (χ^2^ = 79.42, df = 23, *p*<0.001), therefore a random effect model was applied.

**Figure 2:**
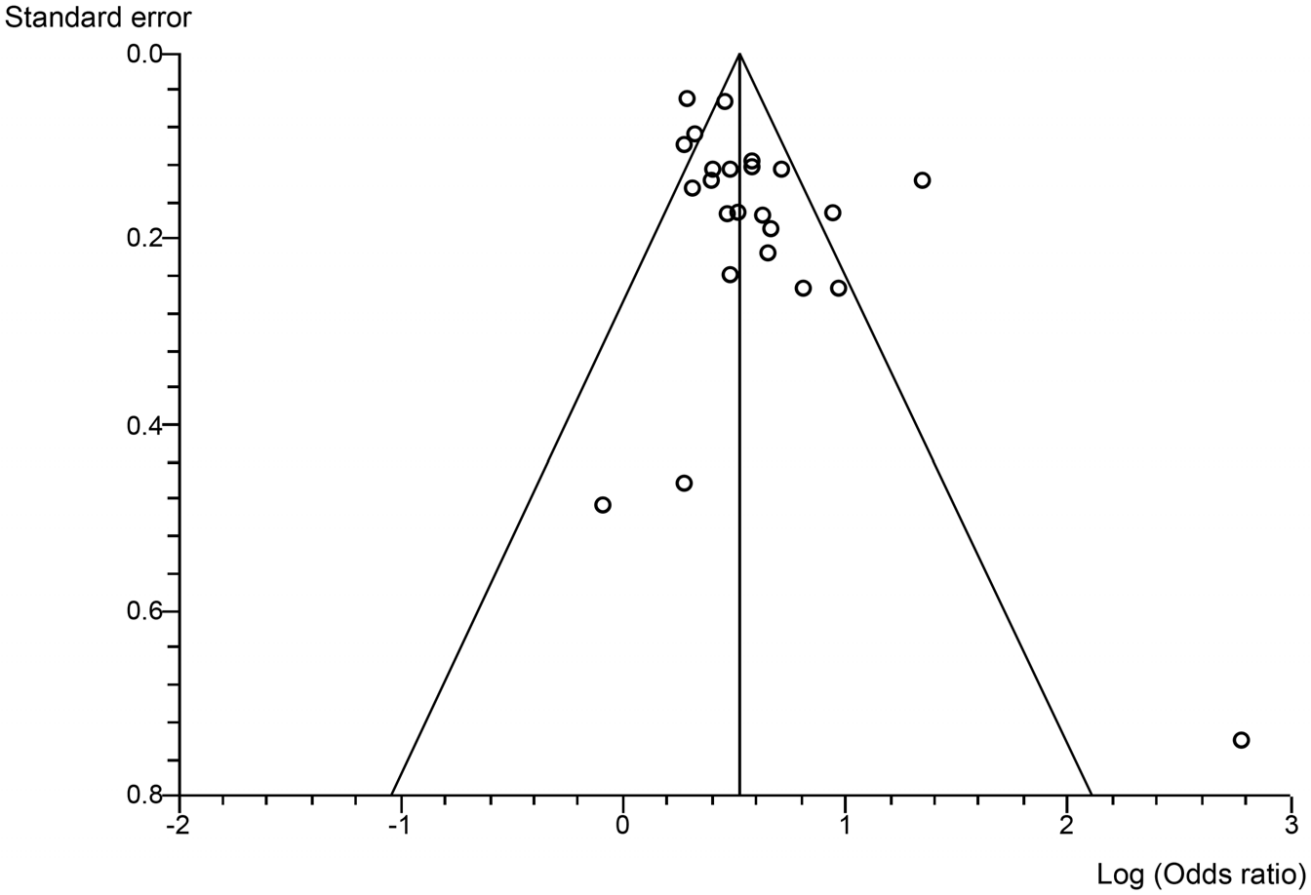
Funnel plot. Funnel plot of published studies in relation to the first meta-analysis (24 studies).

The funnel plot (Figure 2) did not show any publication bias, in accordance with the bias tests: Begg-Mazumdar: Kendall τ = −0.05, *p* = 0.76; Egger: bias = −0.55, *p* = 0.46.

There was not a significant difference between the first meta-analysis and the second one: combined OR = 1.80 with 95%CI = (1.61–2.02) using random effect estimate (Cochran's Q test: χ^2^ = 79.42, df = 22, *p*<0.001).

The studies with a quality score ≥11 were 14. The relationship between *PTPN22* 1858C>T SNP and RA resulted even stronger: OR = 2.01, 95%CI = (1.67–2.43) using random effect model (Cochran's Q test: χ2 = 61.14, df = 13, *p*<0.001).

**Figure 3:**
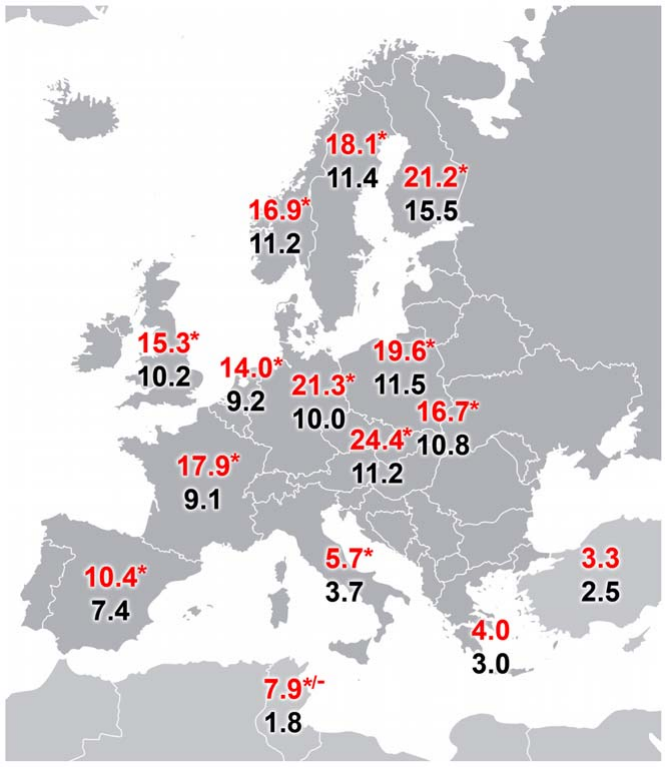
Geographical distribution in Europe. Geographical distribution of the T allele frequency at *PTPN22 rs2476601* SNP in European RA patients (red) and healthy controls (black) [5], [7]–[9], [16]–[37]. The ‘*’ symbol refers to countries in which a statistical significant different distribution of the *PTPN22* T1858 allele among patients and controls was noted. In Tunisia the two existing articles regarding *PTPN22* show opposite findings.

When looking at the T allele frequency in RA patients and controls, we noticed a North-South gradient with higher values in Finland, Germany, Hungary, and lower values in Spain, Italy, Tunisia, Greece and Turkey (Figure 3 [5], [7]–[9], [16]–[37]).

## Discussion

The association between the T1858 allele at *rs2476601* in the *PTPN22* gene and RA has been documented in several cohorts, from the USA as well from Europe [38], [39], though it seems to be less relevant in other continents [40]. When considering the strength of the allele association in the analysis of the European consortium, the relationship arose quite clear; however regarding single countries, data are less clear-cut. As reported in Figure 3, a North-South gradient seems to be present in the distribution of the T1858 allele in both RA patients and controls, as previously remarked by Gregersen *et al.* in some European populations [38].

Furthermore, it is also noteworthy that while in Germany, the frequency of the T1858 allele was significantly higher in RA patients (21.3%) compared to controls (10.0%; with an OR of 2.43) and the association was present irrespective of the presence or absence of anti-CCP and RF [41], in France the European Consortium Group provided evidence for an association of T1858 allele only with RF positive cases but not with RF negative RA patients [36]. In Spain, there was no association with early RA but the association was significant with the anti-CCP positive RA [6].

Genetic differences within European populations have been once more underlined by a recent work of Rodríguez-Rodríguez *et al.* [42]. The authors described the association of another *PTPN22* SNP, the R263Q, with RA in six different Caucasian populations. The 1858C>T SNP was also investigated using mostly previously published data. The T allele of the 1858C>T SNP showed an inhomogeneous distribution among the populations taken into account, with a prevalence of 10.5% in RA patients and 6.8% in controls in Spain, compared to 16.1% and 10.6% respectively in the other countries (Norway, UK, The Netherlands, Germany, New Zealand).

Our data revealed a higher frequency of the T1858 allele in RA Italian patients compared to the controls cohort. On the other hand, the frequency in controls was lower than that observed in France or in Germany and similar to Turkish, Greek and Tunisian populations.

Interestingly, Mediterranean populations are genetically linked by a common history of migrations, like the abiding one of Saracens and Moors. In fact a recent work, estimating the medieval North African contribution over Mediterranean countries through the analysis of the Y chromosome short tandem repeats, suggested a general correlation between historical and genetic data of Iberia, Sicily, Turkey and North Africa [43].

No relationship arose between the C/T-T/T genotypes presence and auto-antibodies positivity. The demonstration of a gain-of-function conferred by the T1858 allele in suppressing TCR (T cell receptor) function in T cells and BCR (B cell receptor) function in B cells raises new hypotheses on the role of tyrosine phosphatases. The T1858 allele might increase the threshold for a persistent activation of both autoreactive T and B cells thus leading to a more defined autoimmune subset of RA [4]. In our study, the trend for an association between the *rs2476601* SNP and the positivity of anti-CCP seems to move towards this direction, though the only conclusion we can formulate with the data at hand is the geographical issue.

In conclusion, the geographical distribution of SNPs in the world, linked to different population origins, should be taken into account in studies regarding genetic associations. Given that specific therapies directed toward Lyp will be available in the near future for various autoimmune diseases [44], there could be clinical-therapeutic consequences as well, and, on these grounds, the approach based on *PTPN22* might be different from North to South.

## Supporting Information

Figure S1 **Electrophoresis gel.** Photo of the electrophoresis gel showing intact *vs.* cleaved PCR-amplified fragments from patients and controls, non-mutated, heterozygous or homozygous for the C>T substitution. A: intact PCR-amplified fragment; B: cleaved fragment from DNA of a patient non-mutated for the *PTPN22 rs2476601* SNP; C: cleaved fragment from DNA of a patient heterozygous for the SNP; D: cleaved fragment from DNA of a patient homozygous for the SNP; E, F and G: same as for B, C and D, but from the DNA of a control subject.
(TIF)

Figure S2 **PRISMA flow diagram.** PRISMA 2009 flow diagram regarding the article selection.
(DOC)

PRISMA Checklist S1 **PRISMA Checklist.** PRISMA 2009 checklist regarding meta-analysis data and their position in the manuscript.
(DOC)
