# Supplementary figures and images for: PTPN22 1858C>T Polymorphism Distribution in Europe and Association with Rheumatoid Arthritis: Case-Control Study and Meta-Analysis
Source: PLoS One. 2011 Sep 16;6(9):e24292. doi: 10.1371/journal.pone.0024292 (PMC3174938; doi:10.1371/journal.pone.0024292)

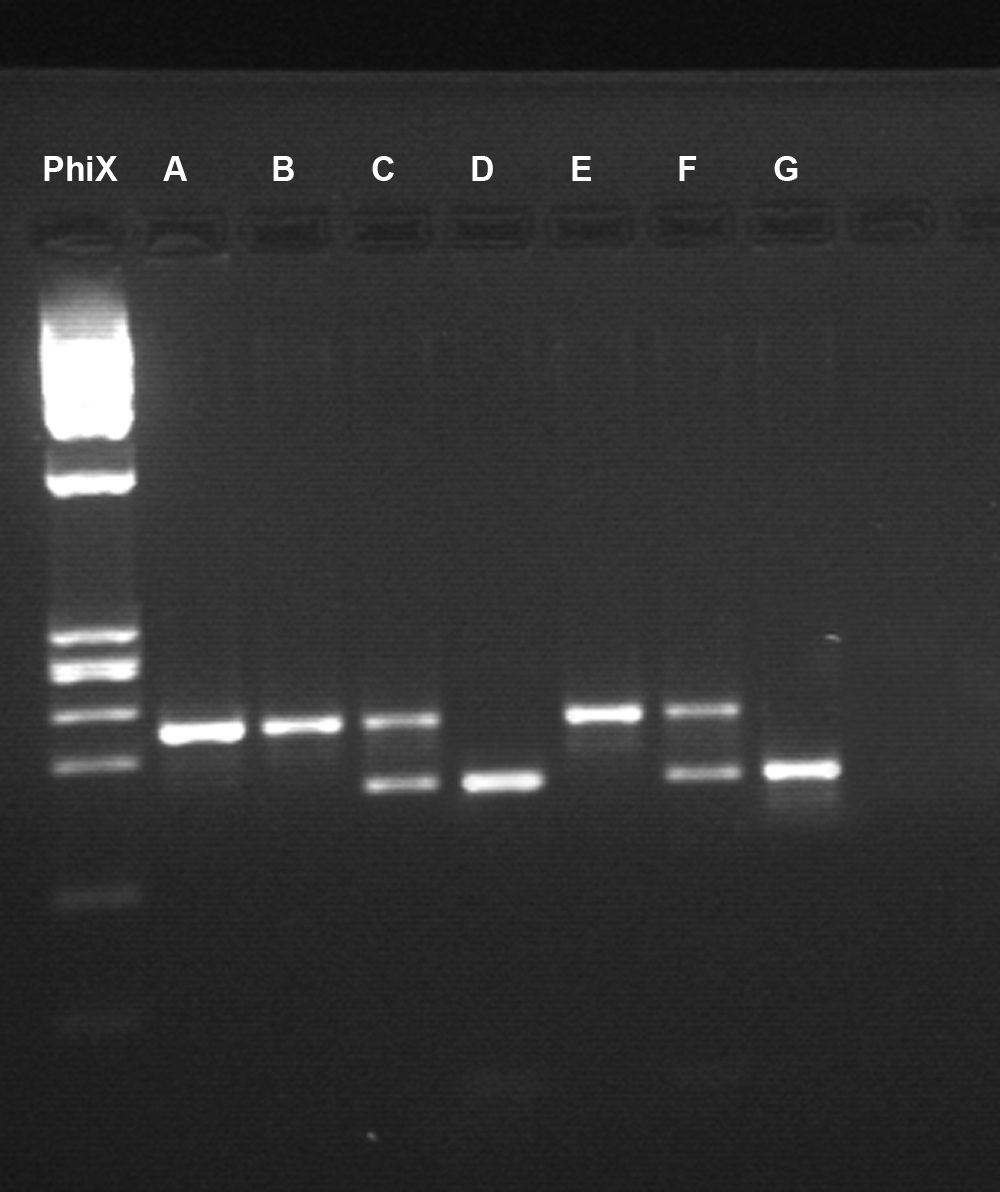

Supplement: Figure S1 — Electrophoresis gel. Photo of the electrophoresis gel showing intact vs. cleaved PCR-amplified fragments from patients and controls, non-mutated, heterozygous or homozygous for the C>T substitution. A: intact PCR-amplified fragment; B: cleaved fragment from DNA of a patient non-mutated for the PTPN22 rs2476601 SNP; C: cleaved fragment from DNA of a patient heterozygous for the SNP; D: cleaved fragment from DNA of a patient homozygous for the SNP; E, F and G: same as for B, C and D, but from the DNA of a control subject. (TIF) [file pone.0024292.s001.tif]
