# Supplementary material for: PTPN22 1858C>T Polymorphism Distribution in Europe and Association with Rheumatoid Arthritis: Case-Control Study and Meta-Analysis
Source: PLoS One. 2011 Sep 16;6(9):e24292. doi: 10.1371/journal.pone.0024292 (PMC3174938; doi:10.1371/journal.pone.0024292)
Supplement: Figure S2 — PRISMA flow diagram. PRISMA 2009 flow diagram regarding the article selection. (DOC) [file pone.0024292.s002.doc]

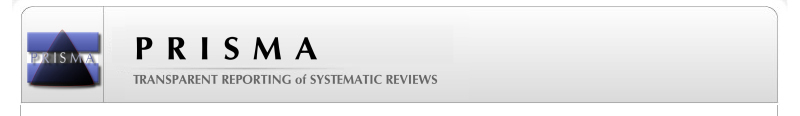
**PRISMA 2009 Flow Diagram**

**Screening**

**Included**

**Eligibility**

**Identification**

Records identified through database searching
(n = 143 )

Additional records identified through other sources
(n = 186 )

Records after duplicates removed
(n = 157 )

Records screened
(n = 157 )

Records excluded
(n = 119 )

Full-text articles assessed for eligibility
(n = 36 )

Full-text articles excluded, with reasons:
Studies that lacked useable information (n=9)

Reviews Articles (n=1)

Repeated data sets (n=2)

Studies with transmission disequilibrium test (n=1)

TOTAL N=13

Studies included in qualitative synthesis
(n = 23 )

Studies included in quantitative synthesis (meta-analysis)
(n = 23 )
